# Antidepressant and Antipsychotic Drug Use and Cancer Risk: Protocol for an Overview of Systematic Reviews and Meta-Analyses

**DOI:** 10.2196/78596

**Published:** 2025-12-23

**Authors:** Joan Vicent Sánchez-Ortí, Jaume Forés-Martos, Vui Doan, Pablo Vicente-Martínez, Diego Macías Saint-Gerons, María Flores-Rodero, Jon Sánchez-Valle, Patricia Correa-Ghisays, Vicent Balanzá-Martínez, Pau Soldevila-Matías, Joan Vila-Francés, Emilio Soria-Olivas, Alfonso Valencia, Rafael Tabarés-Seisdedos

**Affiliations:** 1 Center for Biomedical Research in Mental Health Network (CIBERSAM), Health Institute, Carlos III Madrid Spain; 2 INCLIVA Health Research Institute Valencia Spain; 3 Unit of Psychiatry and Psychological Medicine Department of Medicine University of Valencia Valencia Spain; 4 TMAP - Evaluation Unit in Personal Autonomy, Dependency and Serious Mental Disorders Universitat de València Valencia Spain; 5 Stanford Medicine Stanford, CA United States; 6 Intelligent Data Analysis Laboratory University of Valencia Valencia Spain; 7 Department of Nursery Faculty of Nursery Universidad de Valladolid Valladolid Spain; 8 Barcelona Supercomputing Center Barcelona Spain; 9 Faculty of Psychology University of Valencia Valencia Spain; 10 VALencia Salut Mental i Estigma (VALSME) Research Group, University of Valencia Valencia Spain; 11 Catalan Institution for Research and Advanced Studies Barcelona Spain

**Keywords:** antidepressants, antipsychotics, cancer, drug repurposing, large language models, meta-analysis, systematic review

## Abstract

**Background:**

The relationship between cancer and central nervous system disorders has received increasing attention recently. Consequently, antipsychotics and antidepressants, commonly prescribed for conditions such as depression, bipolar disorder, and schizophrenia, have emerged as potential modulators of subsequent cancer risk. Previous studies have suggested that the use of these medications is associated with a decreased risk of cancer incidence and mortality, making them suitable candidates for drug repurposing. However, the potential therapeutic benefits do not extend to all cancer types, as some data suggest an increased risk for specific tumors.

**Objective:**

This study aims to conduct a comprehensive review of systematic reviews and meta-analyses (review of reviews) that assess whether exposure to antidepressants or antipsychotics influences cancer incidence and mortality.

**Methods:**

To provide a clear overview of this review, we have designed and registered the study protocol. Specifically, we will include systematic reviews and meta-analyses that examine the relationship between previous antipsychotic or antidepressant treatments and the subsequent cancer risk. The primary outcome will be the risk of cancer incidence and mortality (all malignant neoplasms) associated with exposure to psychopharmacological medications. Furthermore, secondary outcomes will include site-specific cancer incidence and mortality (eg, lung cancer). Literature searches will be conducted in multiple electronic databases (from their inception onwards), including PubMed/MEDLINE, Embase, and the Cochrane Database of Systematic Reviews. Three researchers will independently screen all citations, abstracts, and full-text articles. We will perform parallel search, selection, and extraction tasks using a large language model (GPT-4o; OpenAI). Data selection and extraction will involve both human reviewers and GPT-4o, whose performance will be validated through human evaluations. Thus, we will verify whether this type of tool can accelerate or even perform the tasks involved in a systematic review. The risk of bias and the quality of individual studies will be evaluated using appropriate tools. Subsequently, we will extract the summary association measures (eg, pooled relative risk, odds ratio, and hazard ratio) as reported in each included systematic review. Where available, we will summarize subgroup and sensitivity analyses as described by the authors.

**Results:**

Planned searches will be conducted in various electronic databases from their creation until September 2025. No results are available or included in this protocol. The expected results will be published in 2026.

**Conclusions:**

This overview of systematic reviews and meta-analyses will provide an updated synthesis of the cancer risk associated with antipsychotic and antidepressant drugs. Furthermore, this study will examine factors that may explain potential study variations. Ultimately, these findings will be published in a peer-reviewed journal.

**Trial Registration:**

OSF Registries 10.17605/OSF.IO/5ACWH; https://osf.io/5acwh/overview

**International Registered Report Identifier (IRRID):**

DERR1-10.2196/78596

## Introduction

Recently, the relationship between cancer and the nervous system has attracted considerable attention for several reasons. Cancer and neurological and mental disorders are among the leading causes of death and are usually chronic, debilitating disorders with profound human and economic consequences [1-3].

Second, many recent preclinical and clinical studies have investigated the crosstalk between the nervous system and cancer. Notably, the nervous system and psychopharmacological interventions can regulate any aspect of cancer biology, from tumor initiation to progression and metastasis. Conversely, cancer and cancer therapies can influence and remodel the nervous system [4,5]. The importance of this evidence has culminated in the emergence of a new discipline termed “cancer neuroscience” (a review is provided elsewhere [6-8]). However, the absence of references to the reciprocal interactions between cancer and neurological and mental disorders in these reviews is surprising.

Third, several systematic reviews and meta-analyses have suggested that neurological and mental disorders such as schizophrenia, mood disorders, autism spectrum disorders, anorexia nervosa, Alzheimer disease, Parkinson disease, and multiple sclerosis are associated with a significantly increased or decreased risk of various cancers, including melanoma and brain, breast, pancreatic, and lung cancers [9-12]. Although knowledge gaps exist regarding these direct and inverse comorbidities, a cross-diagnostic perspective is required to better understand the nervous system–cancer interactions [13].

Fourth, given the established comorbidities between cancer and neurological and mental disorders, the remaining questions are as follows: What are the underlying causes, and how can they be identified? Many factors are thought to generate dependencies among these diseases. They are linked to the environment, lifestyle, drug treatments, genetic factors, and, more probably, a combination thereof. Antidepressants and antipsychotic drugs have emerged as repurposed treatments in oncology [7]. Drug repurposing involves the development of new indications for the use of existing neuroactive compounds to treat drug-resistant forms of cancer. However, these advantages do not extend to all cancer types, as some antidepressants and antipsychotic drugs contribute to tumor proliferation [14].

Fifth, antidepressants and antipsychotics are among the most commonly prescribed pharmacological treatments worldwide. They are also used off-label and for nonpsychiatric indications [15-17]. The neurochemical targets of these medications have been postulated to modulate both cancer risk and oncological outcomes [18-23]. Consequently, based on these observations, antidepressants and antipsychotics can affect cancer risk and mortality [24,25].

Psychiatric and neurological disorders themselves have been associated with an altered cancer risk, possibly due to shared genetic, behavioral, or lifestyle factors. On the other hand, pharmacological treatments with antidepressants and antipsychotics can independently modulate cancer biology through mechanisms such as apoptosis, immune regulation, and angiogenesis. While individual systematic reviews have examined specific classes of drugs (eg, selective serotonin reuptake inhibitors [SSRIs]) or individual cancer sites (eg, breast or lung cancer), no comprehensive review has synthesized the findings across all classes of antidepressants and antipsychotics and a broad spectrum of oncological outcomes. Addressing this gap provides the novelty and necessity of this study. This study focuses specifically on drug exposure, recognizing that underlying comorbidities act as potential confounding factors.

We hypothesize that exposure to antidepressant and antipsychotic drugs may alter cancer incidence and mortality by modulating oncogenic pathways, such as cell proliferation, apoptosis, and tumor angiogenesis. This theoretical framework underpins the study design and guides data synthesis.

Therefore, this study is an overview of systematic reviews and meta-analyses evaluating the association between antidepressant and antipsychotic drug use and cancer risk and mortality. Further studies are needed to determine whether antidepressant and antipsychotic use has an antineoplastic effect leading to improved oncological outcomes.

## Methods

### Ethical Considerations

Ethical approval is not required for this study, as it is a general review of systematic reviews based solely on previously published aggregate data and therefore does not involve human participants or identifiable personal data. According to guidelines such as the Cochrane Handbook for Systematic Reviews, ethical approval is not typically needed for systematic reviews of published literature.

### Overview of Systematic Reviews (With or Without Meta-Analyses)

An overview of the review protocol has been registered in the Open Science Framework (OSF). It will be conducted following the reporting guidance from the PRISMA-P (Preferred Reporting Items for Systematic Reviews and Meta-Analyses Protocols) statement [26,27] (PRISMA-P checklist is provided in Multimedia Appendix 1 [27]).

This protocol has been assigned the International Registered Report Identifier (IRRID): RR1-10.17605/OSF.IO/5ACWH.

The PICO (Population, Intervention, Comparator, Outcome) question includes people currently receiving psychopharmacological treatment, specifically with antipsychotic or antidepressant medications, including children, adolescents, and adults. The intervention is exposure to antidepressants or antipsychotics, the comparator is unexposed populations, and the outcomes are incidence and mortality of all cancers and localized cancers.

### Criteria for Considering Studies for This Overview of the Review

#### Types of Studies

We will include all systematic reviews (with or without meta-analyses) of observational studies (that is, cohort and case-control studies) and experimental studies (such as randomized controlled clinical trials) focusing on the association between antipsychotic and antidepressant drugs used among children, adolescents, and adults with any central nervous system disorder and cancer risk. We will exclude studies that (1) do not examine the use of antipsychotics and antidepressant drugs, (2) do not assess the outcomes of interest (including, but not limited to, incidence, progression-free survival, disease-free survival, overall survival, and mortality of any cancer type), and (3) include meta-analyses with fewer than 3 original studies. Meta-analyses that include fewer than 3 primary studies will be excluded from the quantitative synthesis to prevent unstable estimates. However, such reviews will be described narratively in the sensitivity analyses, especially when addressing rare cancers. Only peer-reviewed journal articles written in any language will be included. Animal or genetic studies will be excluded. Furthermore, if the same association was examined in 2 or more meta-analyses, associations from different meta-analyses will be considered duplicates. Therefore, the meta-analysis with the largest sample size will be selected to avoid overlap.

We will search registries, preprint servers, and gray literature to ensure comprehensiveness and transparency. Inclusion will be limited to systematic reviews or meta-analyses that report on methods consistent with PRISMA (Preferred Reporting Items for Systematic Reviews and Meta-Analyses) and provide sufficient data for evaluation. Preprint or gray literature reviews will only be included if they meet these minimum information criteria and will be examined in sensitivity analyses (primary analyses will prioritize peer-reviewed publications).

#### Types of Participants

We will include participants who are being treated with antipsychotic and antidepressant medications, regardless of their diagnosis. The inclusion criterion is the current use of these pharmacological agents rather than the diagnostic category defined by the *DSM* (*Diagnostic and Statistical Manual of Mental Disorders*) or *ICD* (*International Classification of Diseases*) criteria [28,29]. We will extract and, where possible, stratify results by indication (psychiatric diagnosis versus nonpsychiatric or off-label use).

#### Types of Interventions

The following antipsychotic groups will be considered for the exposure of interest: (1) first-generation (typical) antipsychotics; (2) second-generation (atypical) antipsychotics; and (3) newer agents, including partial dopamine agonists (D2) such as aripiprazole, brexpiprazole, and cariprazine.

The following antidepressant clusters will be considered for the exposure of interest: SSRIs, serotonin/norepinephrine reuptake inhibitors (SNRIs), monoamine oxidase inhibitors (MAOIs), tricyclic antidepressants (TCAs), serotonin modulators, and atypical antidepressants.

A complete list of antidepressants and antipsychotics is provided in Multimedia Appendix 2.

We will include systematic reviews and meta-analyses regarding antipsychotics and antidepressants approved by the European Medicines Agency (EMA) and the Food and Drug Administration (FDA). We will accept any intervention planned identically in the intervention and control groups except for the drug of interest.

To minimize analytical heterogeneity, results will be categorized by major drug classes: SSRIs, SNRIs, TCA, MAOIs, serotonin modulators, atypical antidepressants, and typical versus atypical antipsychotics. When available, analyses will be stratified by cumulative dose, duration of treatment, and latency between drug exposure and cancer outcomes.

We will extract information on the adjustment of the main confounding factors (psychiatric diagnosis, smoking, alcohol, obesity, and socioeconomic status) and consider them in the interpretation of the evidence.

We will differentiate between studies that evaluate psychiatric indications and those that investigate off-label oncological use, reporting them separately to avoid confusion.

### Types of Outcome Measures

#### Primary Outcomes

The primary outcomes of interest will be cancer incidence and mortality (all malignant neoplasms; *ICD-11* [*International Classification of Diseases, 11th Revision*]: 2A00–2F9Z) associated with exposure to antipsychotic and antidepressant drugs.

Cancers of specific locations will be grouped according to the *ICD* categories (eg, digestive, respiratory, and hematological) to improve reproducibility and transparency.

#### Secondary Outcomes

Secondary outcomes will include site-specific cancers (such as glioblastoma and melanoma).

Studies should explicitly report the number of cancer events in all the treatment groups of interest. Studies that do not present quantitative data on the association between the evaluated interventions and cancer events or provide sufficient data for computing such measures of association will be excluded [30].

### Search Strategy for Identification of Studies

#### Electronic Searches

The following electronic databases will be searched: MEDLINE [31] is available through PubMed (National Library of Medicine), Embase [32] through the Elsevier platform (Elsevier BV), and the Cochrane Database of Systematic Reviews through the Cochrane Library [33].

Sources of gray literature (conference abstracts and theses), preprint servers, and trial registries (ClinicalTrials.gov, WHO ICTRP) will also be searched to minimize publication bias.

A health sciences librarian (DMS-G) will review and refine the electronic search strategies for each database. The final strategies will be peer-reviewed using the Peer Review of Electronic Search Strategies (PRESS) checklist. All drug names have been verified against EMA and FDA lists and corrected for spelling.

We will use validated search filters to exclude animal studies where appropriate. No restrictions will be placed on age or language. A final search filter for “systematic review” or “meta-analysis” will be applied during the selection stage to improve specificity.

We will search these databases from their inception. The research team will design and perform a detailed search strategy. We will use the same search strategy with appropriate adjustments for each database. Search terms will include keywords related to (1) the exposures of interest: “names/cluster of antidepressant drugs,” and “names/cluster of antipsychotics”; (2) the types of studies: “systematic review” and “meta-analysis,” within which will be specified whether the studies are “observational studies” or “randomized and non-randomized studies”; and (iii) the outcome of interest: “cancer.” No restrictions on data will be imposed. Initial literature searches of MEDLINE, Embase, and the Cochrane Database of systematic reviews will begin in October 2024. The last planned search date was September 2025, reflecting the prospective nature of this protocol. This ensures transparency regarding the planned timeline prior to its completion. Multimedia Appendix 3 provides details of the draft search strategies for MEDLINE and Embase. The GPT-4o (OpenAI) model or similar will be used to obtain article titles relevant to this review, following the previously mentioned search criteria. If this artificial intelligence (AI)–driven title identification is not feasible, we will proceed with the items obtained through the conventional search.

AI-assisted screening with GPT-4o–like will be validated by 2 independent human reviewers. Agreement will be measured using sensitivity, specificity, and Cohen κ index. Discrepancies will be resolved by consensus or by the decision of a third reviewer. Ethical considerations and transparency in rapid reproducibility will be documented. The large language model (LLM) will serve as a complement to, not a substitute for, human reviewers.

When conflicting results arise, priority will be given to higher-quality reviews, larger samples, and more recent publications. Contradictions will be explicitly discussed in sensitivity analyses.

#### Searching Other Resources

We will also use the following methods: (1) check reference lists of all systematic reviews and meta-analyses for additional references, (2) conduct complementary searches for the Cochrane Global Mental Health Group’s specialized register [34], and (3) contact the authors of the studies if clarification is needed or if the reported data must be completed.

### Data Collection and Analysis

#### Software Considerations

All analyses will be performed using Stata (version 18; StataCorp LP) [35,36], R (version 4.2.0; R Core Team), and Python (version 3.12.5; Python Software Foundation).

#### Selection of Systematic Reviews

First, a total of 3 researchers, independently and masked, will screen the titles and abstracts of the articles obtained from the initial searches according to the eligibility criteria. Second, the full texts will be examined in detail and screened for eligibility. Third, the references of all considered articles will be manually searched to identify any relevant reports missed during the search strategy. Disagreements will be resolved through discussions, with a fourth researcher consulted if necessary.

The selection phase will be conducted using the articles selected by the LLMs. During this phase, the model will retain articles that meet the proposed criteria and discard those that do not. Once the documents have been selected, we will test the understanding of the subject matter by asking the model to answer a series of questions about the content or evaluate its conclusions or questions about the articles [37,38].

The authors will be contacted in case of ambiguity or missing data. We will identify and exclude duplicates and collate multiple reports from the same study such that each analysis, rather than each report, is the unit of interest in the review.

In the event of overlapping primary studies, we will calculate the corrected covered area (CCA) and prioritize the most comprehensive, recent, and highest-quality review. When contradictory results are presented, we will adopt a hierarchy considering methodological quality, sample size, and publication date. Sensitivity analyses will assess the robustness of the conclusions. After calculating the CCA, duplicate effect sizes will be removed to avoid double counting of primary studies in different reviews.

First, all primary studies cited in each included review will be assigned to a citation matrix (primary study × review), after which we will calculate the CCA to quantify overlap and classify it as low, moderate, high, or very high. For duplicate associations involving the same exposure and cancer outcome across reviews, we will retain the review that best represents the evidence, prioritizing (1) the highest overall confidence according to *A Measurement Tool to Assess Systematic Reviews, Version 2* (AMSTAR-2), followed by (2) the most recent publication date, (3) the largest number of primary studies, and (4) the broadest population and outcome coverage. The combined estimate from the selected review will be used in the main synthesis, with alternative estimates described narratively. Sensitivity analyses will then be conducted by excluding lower-priority reviews to assess the robustness of the conclusions.

#### Data Collection and Management

After the searches have been performed, the items identified in the databases will be imported to a systematic review manager. This will be performed using Rayyan software (Rayyan Systems) [39]. Following the recommendations of the PRISMA 2020 declaration [40], a flowchart showing the details of the included and excluded studies at each stage of the selection process will be provided.

A total of 3 researchers will independently extract data from each included study, and potential conflicts will be resolved through discussion. We will examine the full articles and supplementary materials containing data and analyze the general and methodological characteristics, as well as the study results. In addition, we will review the final versions of articles that are available online. Simultaneously, the same procedure will be conducted independently using an AI system, like OpenAI’s GPT-4o model.

We will use OpenAI’s GPT-4o–like models as an assistance tool under strict governance. The model version, application programming interface (API) parameters, and all prompts will be recorded and stored in OSF. The LLM will be applied to classifying titles or abstracts and prefilling structured extraction forms; however, the gold standard for decisions remains human reviewers. All title or abstract records will be independently reviewed by 2 human reviewers; the LLM will run in parallel for classification, and its results will be compared to human consensus along all records obtained through the searching phase. The model acceptance thresholds for selection are a sensitivity of at least 80% (to identify potentially eligible records) and a Cohen κ of at least 0.6 against human consensus. The exact metrics that will be considered for evaluating AI’s performance are accuracy, sensibility, recall, and *F*_1_-score. To obtain these metrics, the human reviews will be used as ground truth to determine whether AI could have substituted reviewers in this process. For extraction, the accuracy of exact match of key fields must reach at least 90% before machine results are used to prepopulate forms; even then, 2 human reviewers will verify all extracted data. If the thresholds are not met, the LLM will not be used for decision-making. All discrepancies between the LLM and humans will be documented, resolved by consensus or by the decision of a third reviewer, and communicated. The team will not upload identifiable patient data or datasets containing personal information into the model; only bibliographic metadata and anonymized text snippets strictly necessary for selection and extraction will be provided. Use of the model will comply with institutional data protection policies and OpenAI’s data processing terms.

GPT-4o model or a similar model will be used for titles and abstracts (metadata) solely for classification purposes. During full-text selection and data extraction processes, the LLM will only receive anonymized text fragments (such as PICO elements or brief results tables), and only when permitted by data processing agreements and publisher terms. No personally identifiable information, raw patient-level data, or proprietary full PDF files will be fed into the model. Use of the model will comply with institutional data protection guidelines, and will be recorded and archived.

Data extracted with GPT-4o or similar will be compared with independent human extractions for at least 20% of the included reviews. Agreement on key variables (effect sizes, CIs) will be reported, and discrepancies will be resolved by consensus.

Data from each report will be used to construct evidence tables describing the included studies. The standardized data extraction form will contain the following information: first author’s name, year of publication, drug type or cluster (such as fluoxetine or SSRIs), outcome (including melanoma or its subtypes), comparison, the total number of participants, number of cancer events, and number of included studies extracted from each eligible meta-analysis. The study design (randomized controlled trial or cohort studies), number of participants (control and cases), participant characteristics (such as diagnostic criteria, age, sex, years of education, comorbidity, or risk factors), and maximally adjusted effect size will also be extracted.

The data extraction form will undergo 3 pilot tests to refine its consistency before full implementation. It will undergo 3 testing rounds on about 10% of qualifying reviews (minimum of 5). In the pilot test, 2 independent reviewers will extract data; interrater reliability (Cohen κ) will be measured for key fields, and the form will be refined until a κ of 0.75 or higher is reached. Subsequently, all included reviews will be independently extracted by 2 reviewers, with discrepancies resolved via consensus or with a third party’s intervention.

We will extract information on the adjustment for confounding factors described in each review and in the studies included, specifically whether the analyses were adjusted for smoking, alcohol consumption, obesity or BMI, psychiatric diagnosis, socioeconomic status, and other risk factors. We will summarize the degree of adjustment for confounding factors and consider this when interpreting the strength and direction of the observed associations.

This study is an overview of systematic reviews. We will not re-extract primary study data nor perform second-order meta-analyses pooling primary study data. Instead, for each included systematic review or meta-analysis, we will extract the effect measures and statistics as reported (eg, pooled relative risk [RR], odds ratio [OR], hazard ratio [HR], 95% CI, *I*², model type). Results will be synthesized narratively with structured summary tables. When multiple reviews report the same association, we will apply the overlap and priority algorithm to prevent double-counting.

### Risk of Bias and Quality Assessment in Included Reviews

At least 2 researchers will independently assess the risk of bias and the quality of each study using the AMSTAR-2 [41], and any disagreements will be resolved through discussion. The risk of bias in each study will be judged based on each criterion and classified as critically low (more than one critical defect with or without noncritical weaknesses), low (one critical defect with or without noncritical weaknesses), moderate (more than one noncritical weakness), or high (no or one noncritical weakness). The interrater reliability of AMSTAR-2 assessments will be quantified using Cohen κ index to ensure transparency and reproducibility.

We will quantify the overlap of primary studies in eligible systematic reviews by calculating the CCA using the number of eligible systematic reviews, and all included primary studies [42]. We will classify the degree of overlap according to the percentage as slight (0%-5%), moderate (6%-10%), high (11%-15%), or very high (>15%).

We will also evaluate the strength of the evidence for each outcome presented in the overview using the GRADE (Grading of Recommendations, Assessment, Development, and Evaluation) classification system. The evidence classifications are divided into “high,” “moderate,” “low,” and “very low” quality to make recommendations [43].

GRADE certainty ratings will incorporate AMSTAR-2 quality, and will be downgraded when systematic reviews have methodological shortcomings. This approach ensures that the synthesis of evidence reflects both quantitative results and methodological rigor. Reviews rated as “critically low” will not contribute to the pooled synthesis but will be described narratively. AMSTAR-2 ratings will be used directly for GRADE assessments, and downgrading will be applied when reviews show methodological deficiencies.

### Methods for Evidence Synthesis

#### Measures of Treatment Effect

The effect size included in each meta-analysis will be extracted, and 95% CIs will be used. Where available, we will extract relative risk and HRs with 95% CIs, ORs, and standardized incidence/mortality (30). Statistical significance will be set at *P*<.05. Effect sizes derived from the models adjusted for the maximum number of potentially confounding variables will be selected.

Contradictory results will be examined, prioritizing higher-quality and more comprehensive reviews. We will also perform sensitivity analyses to assess the robustness of conclusions when conflicting results are presented.

We will not recalculate the pooled estimates from the primary studies. Effect sizes will be extracted as reported in the included systematic reviews and meta-analyses. Narrative synthesis will be used when quantitative pooling is not appropriate. When multiple effect sizes are reported for overlapping results, we will extract only the most comprehensive or adjusted estimate to avoid double counting.

We will not perform second-order meta-analyses of pooled estimates. Instead, we will summarize the results narratively and emphasize methodological differences between reviews.

#### Assessment of Heterogeneity

The clinical and methodological diversity of participants, interventions, outcomes, and characteristics of the included studies will be assessed to determine the appropriateness of the meta-analysis.

Statistical heterogeneity between studies will be assessed by visually inspecting forest plots and using *I*^2^ statistics. The interpretation of an *I*^2^ value of 0%-40% may not be necessary, 30%-60% may represent “moderate” heterogeneity, 50%-90% may represent “substantial” heterogeneity, and 75%-100% may represent “considerable” heterogeneity [44,45].

All substantial heterogeneity identified will be reported and investigated following the recommendations of the Cochrane Handbook for Systematic Reviews of Interventions [46].

We will extract reported effect estimates and their CIs as presented in each included systematic review or meta-analysis. Harmonization or recalculation of effect measures will not be conducted. When reviews provide subgroup or sensitivity analyses, we will summarize their findings narratively. No second-order meta-analysis or meta-regression will be performed. Reported sources of heterogeneity (as described in each review) will be summarized qualitatively [47].

To limit the risk of post-hoc bias, analyses will be hypothesis-driven and adjusted for multiple testing.

#### Assessment of Reporting Biases

We will not recalculate the pooled estimates from primary studies or perform second-order meta-analyses or meta-regressions on their data. Instead, we will extract the pooled effect estimates and heterogeneity statistics (*I*²) [48,49] as reported in each included review and compile them into structured tables. All meta-regressions or subgroup analyses mentioned in the included reviews will be extracted and explained.

#### Additional Analysis

If sufficient studies are identified and data points are available, the potential sources of heterogeneity will be further investigated using subgroup analyses according to clinical and methodological covariates. We will explore the sources of heterogeneity using the following a priori subgroup hypotheses: (1) study design (observational vs experimental), (2) different drug clusters (antipsychotics vs antidepressants), and (3) type of cancer measure (incidence vs mortality).

To mitigate heterogeneity, subgroup analyses and narrative synthesis (Synthesis Without Meta-Analysis guidelines) will be applied when grouping is not appropriate. Analyses will be stratified by age (children and adolescents vs adults) to account for differences in cancer risk profiles.

The final manuscript will outline and report any amendments made to the study protocol. The results will be disseminated through conference presentations and publications in peer-reviewed journals. All data underlying the findings reported in the final manuscript will be deposited in a cross-disciplinary public repository such as the OSF [50].

## Results

**Figure 1:**
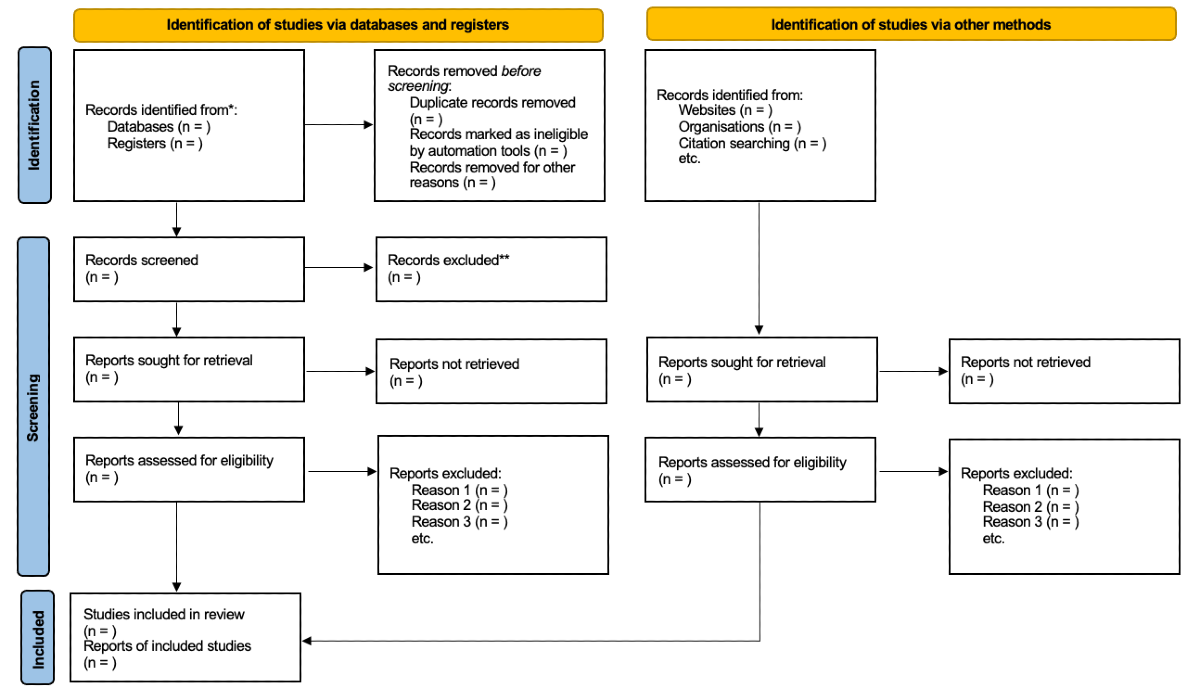
PRISMA (Preferred Reporting Items for Systematic Reviews and Meta-Analyses) 2020 flow diagram. *Consider, if feasible to do so, reporting the number of records identified from each database or register searched (rather than the total number across all databases/registers). **If automation tools were used, indicate how many records were excluded by a human and how many were excluded by automation tools.

Searches will be carried out as outlined in Methods, and findings will be reported in a later manuscript. The final planned search date is September 2025. Results will be shared in peer-reviewed journals. The expected results will be published in 2026. We will present a PRISMA 2020 flow diagram summarizing the selection process (Figure 1; [40]).

## Discussion

### Anticipated Main Findings

This systematic review and meta-analysis aims to provide the most comprehensive synthesis to date on the relationship between antidepressant and antipsychotic use and cancer incidence and mortality. Based on previous evidence, we expect that the results will show a complex and varied picture [1,12-14]. Some classes of psychotropic drugs, such as SSRIs and certain antipsychotics, may exhibit potential protective effects against specific types of cancer, possibly through mechanisms such as inducing apoptosis, modulating the immune system, and inhibiting angiogenesis. Conversely, other agents might be associated with an increased risk of tumor development or progression, reflecting the pharmacodynamic diversity among different drugs. The overview is also likely to emphasize the challenge of establishing causal inference, given that psychiatric disorders themselves, lifestyle factors, and socioeconomic variables act as significant confounders. By integrating data from a wide range of systematic reviews, this study aims to identify where strong evidence exists, where contradictions remain, and where methodological variability limits confidence in the conclusions.

### Comparison With Previous Work

Previous individual systematic reviews have explored the links between the use of antidepressants or antipsychotics and specific types of cancer, such as breast, lung, or liver cancer [51-55]. Some suggest there are inverse relationships, while others report no significant effect or even an increased risk [56]. However, no previous work has systematically combined this evidence across both drug classes and a wide range of cancers. Compared to earlier reviews, this study will expand the scope by including more systematic reviews, using standard assessment tools like AMSTAR-2 and GRADE, and measuring the overlap between the meta-analyses. In this way, the review will clarify whether the associations are consistent, whether they are observed across different study designs and populations, and how much credibility should be given to results that may be biased by methodological flaws or industry ties. By integrating our synthesis into emerging fields such as “cancer neuroscience,” this review will also place the potential for repurposing psychopharmacological drugs within a broader biomedical framework.

### Strengths and Limitations

One of the key strengths of this review is its methodological rigor, which includes a protocol registered in OSF, systematic searches across multiple databases, the inclusion of publications in both English and other languages, and double screening assisted by humans and AI to ensure reproducibility and efficiency. The use of validated instruments, such as AMSTAR-2 for quality assessment and GRADE for certainty of evidence, further improves reliability. Another strength is the explicit consideration of heterogeneity by drug class, cancer type, treatment duration, and cumulative exposure, enabling more nuanced conclusions.

Nevertheless, it must be recognized that there are notable limitations. Overall systematic reviews are constrained by the quality and scope of the included evidence, and the risk of double counting primary studies in meta-analyses remains a concern, despite plans to compute the corrected area under the curve. Many of the included reviews are based on observational studies, which are inherently susceptible to residual confounding from clinical and lifestyle factors such as smoking, obesity, and psychiatric diagnoses. Publication bias, along with selective reporting, may further distort the observed associations. These limitations could impact both the internal validity and wider applicability of the results, particularly when applied to diverse populations or to oncological uses not indicated in the summary of product characteristics of psychopharmacological agents.

### Future Directions

The findings of this review will not offer conclusive causal answers but will serve as a guide for future research. Specifically, well-designed longitudinal studies and randomized controlled trials are necessary to distinguish the effects of drugs from the underlying cancer risk linked to psychiatric disorders. Future research should also include precision medicine approaches, investigating whether genetic or molecular markers influence the impact of exposure to antidepressants and antipsychotics on cancer outcomes. Particular focus should be given to potential protective mechanisms, such as immune regulation, and to identifying high-risk interactions between drugs and cancer. Collaboration across disciplines—such as oncologists, psychiatrists, neuroscientists, and pharmacologists—will be crucial to translating these findings into clinical practice.

### Dissemination Plan

Dissemination will go beyond traditional academic channels. Results will be published in peer-reviewed journals and presented at international scientific conferences. To ensure transparency and reproducibility, all underlying data and analytical code will be uploaded to the OSF. In addition to the academic audience, summaries tailored for clinicians, patient associations, and policymakers will be developed to enhance the practical impact of the findings. Where appropriate, communication materials designed for nonspecialist audiences, including patients and the general public, will be made available to raise awareness of the potential oncological implications of widely prescribed psychopharmacological agents.

### Conclusion

This study protocol outlines a comprehensive and methodologically rigorous approach to synthesizing evidence on the link between antidepressant and antipsychotic use and cancer risk and mortality. Although the expected results will be limited by the heterogeneity and shortcomings of the current literature, the overview will offer vital insights into the potential for drug repurposing in oncology, identify gaps in existing knowledge, and establish a foundation for future research and policy development in the emerging field of cancer neuroscience.

## Appendix

Multimedia Appendix 1 PRISMA-P Checklist.

Multimedia Appendix 2 Table S1. Complete list of antidepressants and antipsychotics.

Multimedia Appendix 3 Key terms for MEDLINE and Embase.

## Abbreviations

AI: artificial intelligence
AMSTAR-2: A Measurement Tool to Assess Systematic Reviews, Version 2
API: application programming interface
CCA: corrected covered area
DSM: Diagnostic and Statistical Manual of Mental Disorders
EMA: European Medicines Agency
FDA: Food and Drug Administration
GRADE: Grading of Recommendations, Assessment, Development, and Evaluation
HR: hazard ratio
ICD: International Classification of Diseases
ICD-11: International Classification of Diseases, 11th Revision
IRRID: International Registered Report Identifier
LLM: large language model
MAOI: monoamine oxidase inhibitor
OR: odds ratio
OSF: Open Science Framework
PICO: Population, Intervention, Comparator, Outcome
PRESS: Peer Review of Electronic Search Strategies
PRISMA: Preferred Reporting Items for Systematic Reviews and Meta-Analyses
PRISMA-P: Preferred Reporting Items for Systematic Reviews and Meta-Analyses Protocols
RR: relative risk
SNRI: serotonin/norepinephrine reuptake inhibitor
SSRI: selective serotonin reuptake inhibitor
TCA: tricyclic antidepressant

## Data Availability

All data generated or analyzed during this study (including extracted data tables, analysis scripts, and LLM prompts and outputs used for validation) will be deposited in the Open Science Framework (OSF) under the project record associated with this protocol. Any results that cannot be shared publicly for legal or copyright reasons will be made available upon reasonable request to the corresponding author, subject to approval by the publisher or licensor.
