# Supplementary material for: Antidepressant and Antipsychotic Drug Use and Cancer Risk: Protocol for an Overview of Systematic Reviews and Meta-Analyses
Source: JMIR Res Protoc. 2025 Dec 23;14:e78596. doi: 10.2196/78596 (PMC12775755; doi:10.2196/78596)
Supplement: Multimedia Appendix 2 [file resprot_v14i1e78596_app2.docx]

**Table S1.** Complete list of antidepressants and antipsychotics.

| - First-generation (typical) antipsychotics [approved by the European Medicines Agency (EMA) and/or the U.S. Food and Drug Administration (FDA)]: Amisulpride (Solian®), Clotiapine (Etumina®), Chlorpromazine (Largactil®, Thorazine®), Fluphenazine (Modecate®, Prolixin®), Haloperidol (Haldol®), Levomepromazine (Sinogan®), Loxapine (Adasuve®), Perphenazine (Decentan®, Trilafon®), Periciazine (Nemactil®), Pimozide (Orap®), Pipotiazine (Lonseren®), Sulpiride (Dogmatil®, Digton®, Guastil®, Lebopride®, Psicocen®, Teravil®), Tiapride (Tiapridal®), Thioridazine (Mellaril®), Thiothixene (Navane®), Tioproperazine (Majeptil®), Trifluoperazine (Eskazine®, Stelazine®), and Zuclopentixol (Cisordinol®, Clopixol®). - Second-generation (atypical) antipsychotics [approved by the EMA and/or FDA]: Aripiprazole (Abilify®), Asenapine (Saphris®), Clozapine (Clozaril®, FazaClo®, Leponex®), Iloperidone (Fanapt®), Lurasidone (Latuda®), Olanzapine (Zyprexa®), Paliperidone (Invega®), Quetiapine (Seroquel®), Risperidone (Risperdal®, Arketin®, Diaforin®, Risfarmal®), Sertindole (Serdolect®), and Ziprasidone (Geodon®, Zeldox®). - Newer agents antipsychotics [approved by the EMA and/or FDA]: Brexpiprazole (Rxulti®) and Cariprazine (Reagila®). - Selective Serotonin Reuptake Inhibitors (SSRIs) [approved by the EMA and/or FDA]: Citalopram (Calton®, Citalvir®, Prisdal®, Relapaz®, Seregra®, Seropram®), Escitalopram (Cipralex®, Celexa®, Lexapro®, Esertia®), Fluoxetine (Prozac®, Adofen®, Luramon®, Reneuron®), Fluvoxamine (Dumirox®), Paroxetine (Seroxat®, Arapaxel®, Casbol®, Daparox®, Frosinor®, Motivan®, Xetin®), Sertraline (Besitran®, Aremis®), Viloxazine (Qelbree®), and Zimelidine (Zelmid®). - Serotonin–Norepinephrine Reuptake Inhibitors (SNRIs) [approved by the EMA and/or FDA]: Atomoxetine (Strattera®), Desvenlafaxine (Pristiq®, Enzude®), Duloxetine (Cymbalta®, Xeristar®), Lofepramine (Gamanil®, Lomont®, Tymelyt®), Levomilnacipran (Fetzima®), Milnacipran (Savella®), Nomifensine (Merital®), Reboxetine (Edronax®, Davedax®, Norebox®, Solvex®), and Venlafaxine (Zarelis®, Vandral®, Dobupal®, Venlabrain®, Venlapine®, Venlamylan®, Arafaxina®, Conervin®, Dislaven®, Flaxen®, Levest®). - Monoamine Oxidase Inhibitors (MAOIs) [approved by the EMA and/or FDA]: Isocarboxazid (Marplan®), Moclobemide (Manerix®), Phenelzine (Nardil®), Pirlindole (Lifril®, Pyrazidol®), Selegiline (Plurimen®), and Tranylcypromine (Parnate®). - Tricyclic Antidepressants (TCAs) [approved by the EMA and/or FDA]: Amitriptyline (Elavil®, Tryptizol®, Deprelio®), Amoxapine (Asendin®, Asendis®, Defanyl®, Demolox®, Moxadil®), Clomipramine (Anafranil®), Desipramine (Norpramin®, Pertofrane®), Dosulepine (Prothiaden®, Thaden®), Doxepin (Silenor®), Imipramine (Tofranil®), Maprotiline (Ludiomil®), Melitracen (Deanxit®), Nortriptyline (Martimil®, Paxtibi®), Protriptyline (Vivactil®), Tianeptine (Stablon®, Coaxil®, Tatinol®, Tianeurax®, Salymbra®), and Trimipramine (Surmontil®). - Serotonin modulators and related agents [approved by the EMA and/or FDA]: Nefazodone (Serzone®), Oxitriptan (Cincofarm®, Levothym®, Levotonine®, Oxyfan®, Telesol®, Tript-OH®, Triptum®), Trazodone (Deprax®), Tryptophan (APO-Tryptophan®, TEVA-Tryptophan®, DOM-Tryptophan®, Tryptan®), Vilazodone (Viibryd®), and Vortioxetine (Brintellix®). - Atypical antidepressants and other novel agents [approved by the EMA and/or FDA]: Agomelatine (Valdoxan®, Thymanax®), Amineptine (Survector®), Brexanolone (Zulresso®), Bupropion (Zyban®, Wellbutrin®), Esketamine (Spravato®), Lumateperone (Caplyta®), Mianserine (Lantanon®), and Mirtazapine (Rexer®, Vastat®, Afloyan®). |
| --- |
