# Supplementary material for: Antidepressant and Antipsychotic Drug Use and Cancer Risk: Protocol for an Overview of Systematic Reviews and Meta-Analyses
Source: JMIR Res Protoc. 2025 Dec 23;14:e78596. doi: 10.2196/78596 (PMC12775755; doi:10.2196/78596)
Supplement: Multimedia Appendix 3 [file resprot_v14i1e78596_app3.docx]

| **Additional file**. Key terms for MEDLINE and EMBASE | | |  |
| --- | --- | --- | --- |
| **Search** | | **Query** |  |
| #1 | **Antidepressant drugs** | “Citalopram”[tiab] OR “Escitalopram”[tiab] OR “Fluoxetine”[tiab] OR “Fluvoxamine”[tiab] OR “Paroxetine”[tiab] OR “Sertraline”[tiab] OR “Viloxazine”[tiab] OR “Zimelidine”[tiab] OR “Atomoxetine”[tiab] OR “Desvenlafaxine”[tiab] OR “Duloxetine”[tiab] OR “Lofepramine”[tiab] OR “Levomilnacipran”[tiab] OR “Milnacipran”[tiab] OR “Nomifensine”[tiab] OR “Reboxetine”[tiab] OR “Venlafaxine”[tiab] OR “Isocarboxazid”[tiab] OR “Moclobemide”[tiab] OR “Phenelzine”[tiab] OR “Pirlindole”[tiab] OR “Selegiline”[tiab] OR “Tranylcypromine”[tiab] OR “Amitriptyline”[tiab] OR “Amoxapine”[tiab] OR “Clomipramine”[tiab] OR “Desipramine”[tiab] OR “Dosulepine”[tiab] OR “Doxepin”[tiab] OR “Imipramine”[tiab] OR “Maprotiline”[tiab] OR “Melitracen”[tiab] OR “Nortriptyline”[tiab] OR “Protriptyline”[tiab] OR “Tianeptine”[tiab] OR “Trimipramine”[tiab] OR “Nefazodone”[tiab] OR “Oxitriptan”[tiab] OR “Trazodone”[tiab] OR “Tryptophan”[tiab] OR “Vilazodone”[tiab] OR “Vortioxetine”[tiab] OR “Agomelatine”[tiab] OR “Amineptine”[tiab] OR “Brexanolone”[tiab] OR “Bupropion”[tiab] OR “Esketamine”[tiab] OR “Lumateperone”[tiab] OR “Mianserine”[tiab] OR “Mirtazapine”[tiab] |  |
| #2 | **Antipsychotic drugs** | “Amisulpride”[tiab] OR “Clotiapine”[tiab] OR “Chlorpromazine”[tiab] OR “Fluphenazine”[tiab] OR “Haloperidol”[tiab] OR “Levomepromazine”[tiab] OR “Loxapine”[tiab] OR “Perphenazine”[tiab] OR “Periciazine”[tiab] OR “Pimozide”[tiab] OR “Pipotiazine”[tiab] OR “Sulpiride”[tiab] OR “Tiapride”[tiab] OR “Thioridazine”[tiab] OR “Thiothixene”[tiab] OR “Tioproperazine”[tiab] OR “Trifluoperazine”[tiab] OR “Zuclopentixol”[tiab] OR “Aripiprazole”[tiab] OR “Asenapine”[tiab] OR “Clozapine”[tiab] OR “Iloperidone”[tiab] OR “Lurasidone”[tiab] OR “Olanzapine”[tiab] OR “Paliperidone”[tiab] OR “Quetiapine”[tiab] OR “Risperidone”[tiab] OR “Sertindole”[tiab] OR “Ziprasidone”[tiab] OR “Brexpiprazole”[tiab] OR “Cariprazine”[tiab] |  |
| #3 | **Cancer/neoplasm terms** | “cancer”[All Fields] OR “cancers”[All Fields] OR “cancerous”[All Fields] OR “malignant”[All Fields] OR “malignancy”[All Fields] OR “neoplasm”[All Fields] OR “neoplasms”[MeSH Terms] OR “tumor”[All Fields] OR “tumors”[All Fields] OR “tumour”[All Fields] OR “tumours”[All Fields] OR “carcinoma”[All Fields] OR “sarcoma”[All Fields] OR “leukemia”[All Fields] OR “lymphoma”[All Fields] OR “melanoma”[All Fields] |  |
| #4 | **Combined ADs and APs terms** | #1 AND #2 | |
| #5 | **Combined with cancer-related outcomes** | #4 AND #3 | |
| #6 | **Limit to systematic reviews or meta-analyses** | #5 AND (“systematic review”[tiab] OR “meta-analysis”[Publication Type] OR “review”[tiab]) | |
